# Automated Classification of Physiologic, Glaucomatous, and Glaucoma-Suspected Optic Discs Using Machine Learning

**DOI:** 10.3390/diagnostics14111073

**Published:** 2024-05-22

**Authors:** Raphael Diener, Alexander W. Renz, Florian Eckhard, Helmar Segbert, Nicole Eter, Arnim Malcherek, Julia Biermann

**Affiliations:** 1 Department of Ophthalmology, University of Muenster Medical Center, 48149 Muenster, Germany; helmar.segbert@gmx.de (H.S.); nicole.eter@ukmuenster.de (N.E.); julia.biermann2017@gmail.com (J.B.); 2 Department of Informatics, University of Applied Sciences Darmstadt, 64295 Darmstadt, Germany; a.renz@proventa.de (A.W.R.); arnim.malcherek@h-da.de (A.M.); 3 Department of Informatics, Technical University of Munich, 80333 Munich, Germany; florian.eckhard@tum.de

**Keywords:** machine learning, glaucoma, glaucoma suspects, data annotation, ground truth

## Abstract

In order to generate a machine learning algorithm (MLA) that can support ophthalmologists with the diagnosis of glaucoma, a carefully selected dataset that is based on clinically confirmed glaucoma patients as well as borderline cases (e.g., patients with suspected glaucoma) is required. The clinical annotation of datasets is usually performed at the expense of the data volume, which results in poorer algorithm performance. This study aimed to evaluate the application of an MLA for the automated classification of physiological optic discs (PODs), glaucomatous optic discs (GODs), and glaucoma-suspected optic discs (GSODs). Annotation of the data to the three groups was based on the diagnosis made in clinical practice by a glaucoma specialist. Color fundus photographs and 14 types of metadata (including visual field testing, retinal nerve fiber layer thickness, and cup–disc ratio) of 1168 eyes from 584 patients (POD = 321, GOD = 336, GSOD = 310) were used for the study. Machine learning (ML) was performed in the first step with the color fundus photographs only and in the second step with the images and metadata. Sensitivity, specificity, and accuracy of the classification of GSOD vs. GOD and POD vs. GOD were evaluated. Classification of GOD vs. GSOD and GOD vs. POD performed in the first step had AUCs of 0.84 and 0.88, respectively. By combining the images and metadata, the AUCs increased to 0.92 and 0.99, respectively. By combining images and metadata, excellent performance of the MLA can be achieved despite having only a small amount of data, thus supporting ophthalmologists with glaucoma diagnosis.

## 1. Introduction

Glaucoma is characterized by the loss of retinal ganglion cells, which results in structural changes to the optic disc and progressive visual field (VF) defects [1,2]. Multimodal imaging of the optic nerve head (ONH), such as optical coherence tomography (OCT) and VF testing, allows for early identification of structural damage so that serious visual impairments can be prevented by early initiation of treatment [3,4,5,6]. However, in contrast, to manifest glaucoma with a classic VF defect, the distinction between healthy and diseased discs can be challenging in glaucoma suspected or abnormal optic discs, especially when large optic disc cupping or areas are present [7,8,9,10], which is one reason for referral to a tertiary eye care center.

Since artificial intelligence (AI), specifically machine learning (ML), aims to make accurate predictions on new unknown data by training on data patterns, it has been applied in the field of glaucoma, in which early diagnosis and rapid initiation of therapy could halt progression [11]. The hope is that an automated ML or deep learning (DL) algorithm will accurately differentiate between healthy and glaucomatous discs and thus support ophthalmologists in clinical practice, especially for challenging cases. Thus far, there has been significant progress in the development of algorithms that can be used for glaucoma screening [12,13,14].

However, despite the progress that has been made in developing AI strategies for glaucoma diagnosis, significant hurdles still need to be overcome [15] before these advances can be translated into clinical practice. In order to generate a machine learning algorithm (MLA) that can support ophthalmologists in the diagnosis of glaucoma, a carefully selected dataset that is based on clinically confirmed glaucoma patients is required. The process of labeling or tagging data with information (e.g., glaucoma patient) that makes it understandable and usable for ML and AI systems is called data annotation. Since there is no single test procedure with sufficient sensitivity and specificity, a detailed, clinical examination is necessary for the diagnosis of glaucoma [6]. Therefore, generating a dataset labeled with a correct diagnosis, known as the “ground truth”, is both challenging and time consuming. As any supervised ML or DL approach is dependent on the ground truth as its reference standard, misclassification could lead to a bias in the trained algorithm. For example, although the algorithm could have a high performance and could supposedly distinguish between healthy and sick eyes, this would not correspond to reality if the annotated data used for the training were already misclassified.

Popular DL and ML inputs are structural and functional imaging tests: fundus images and OCT [16,17,18]. Fundus imaging alone for glaucoma diagnosis is unreliable. The Baltimore Eye Study assessed fundus imaging features independently for glaucoma screening and reported a sensitivity of 52% and specificity of 85% [19]. As image acquisition is portable and low cost and is often routinely collected in a standard fashion, it is possible to compile large training datasets that are optimal for DL algorithms [20,21] at the expense of the ground truth.

Compared to fundus imaging, spectral domain optical coherence tomography (SD-OCT) is more accurate in diagnosing glaucoma, with a sensitivity as high as 89% and a specificity of 95% [22]. The diagnosis relies on an accurate segmentation of retinal layers, which frequently fails and requires time-consuming post-processing, which often leads to a reduction in the amount of data available. Thus, datasets with a high ground truth usually come at the cost of data volume.

It is therefore important to generate a sufficiently large dataset to achieve robust performance of the algorithm and prevent overfitting, which occurs when a model learns to fit the training data too closely, capturing noise or irrelevant patterns, which leads to poor performance on unseen or test data [23]. In order for an algorithm to support experienced ophthalmologists in clinical practice, the dataset should also correspond as closely as possible to the real world and should include not only diseased and healthy patients but also patients for whom glaucoma is suspected in the first place. Generating a group of patients with suspected glaucoma is particularly difficult, as these are patients with abnormalities such as optic disc excavation but with no VF defects. A detailed clinical examination of the patients included is therefore necessary [24,25,26].

The main objectives of this work are to address these mentioned hurdles using a dataset consisting of carefully selected and clinically annotated physiologic optic discs (PODs), glaucoma-suspected optic discs (GSODs), and glaucomatous optic discs (GODs), and present the results of an ML algorithm. To overcome the problem of a small dataset, the dataset was artificially enlarged using data augmentation. In the second step, the algorithm was trained with both fundus images and additional patient metadata.

## 2. Materials and Methods

### 2.1. Data Acquisition and Ground Truth Labeling

The dataset used included 1168 eyes of 584 carefully selected patients who underwent consultation at the University Eye Hospital Münster between January 2018 and December 2020. Patients were retrospectively analyzed and assigned to one of the three groups based on their clinical examination findings as well as the assessment of a glaucoma expert or consultant (Table 1).

Group 1—GODs included 336 eyes from patients with GOD cupping with VF defects, a history of intraocular pressure (IOP) above 24 mmHg, the need for anti-glaucomatous therapy, an onset of the disease in adulthood, an open and inconspicuous chamber angle, and the absence of other causes of secondary open-angle glaucoma. The diagnosis was confirmed in clinical practice by a glaucoma expert from our department.

Group 2—GSODs included 310 eyes from patients who were referred to our institute by general ophthalmologists as glaucoma suspects based on the optic disc appearance and who were later judged as normal by glaucoma experts at our institute. By definition, none of the glaucoma suspects received treatment for glaucoma or ocular hypertension nor had a history of IOP above 21 mmHg.

Group 3—PODs included 321 eyes from patients with no suspicious findings of glaucoma or other ocular diseases who received all examinations due to a diseased contralateral eye (e.g., anterior ischemic optic neuropathy) or drug monitoring. This group also contained eyes from patients with non-organic visual loss who were later diagnosed as healthy based on MRI of the head or VEP.

All patients received a comprehensive clinical examination, which included a detailed medical history, refraction of the eye, best corrected visual acuity measurement, slit-lamp bio microscopy, Goldmann applanation tonometry, and indirect ophthalmoscopy of the ONH. All eyes had structural measures of the ONH as obtained via fundus photography (Visucam 500, Carl Zeiss Meditec AG, Jena, Germany), confocal scanning laser ophthalmoscopy (Heidelberg Retina Tomograph, Heidelberg Engineering GmbH, Heidelberg, Germany), and SD-OCT (Spectralis^®^, Heidelberg Engineering GmbH, Heidelberg, Germany). VF analysis was performed with an automated Humphrey Visual Field Analyzer II (HFA II, model 750; Carl Zeiss Meditec AG, Jena, Germany) using the standard program of the 30-2 Swedish interactive threshold algorithm (SITA fast). Exclusion criteria were a history of optic neuropathies or other diseases that affected the VF other than glaucoma, presence of any media opacities that prevented good quality optic disc photographs, Heidelberg Retina Tomograph (HRT) and SD-OCT imaging, or incomplete data.

### 2.2. Data Processing

After the pseudonymization of every patient, fundus images for each eye were exported. The following fourteen (1–14) metadata were exported for every patient. With the help of a ruler, the horizontal (1) and vertical (2) cup–disk ratio (CDR) was determined based on the fundus images. To do so, the size of the cup was divided by the size of the disc. The mean deviation (MD) of the standard automated perimetry (SAP) (3) and the spherical equivalent (4) were calculated for every eye by adding the sum of the sphere power to half of the cylinder power. Furthermore, the global Bruch’s membrane opening minimal rim width (BMO-MRW) (5); global retinal nerve fiber layer (RNFL) thickness (6); and the RNFL in six subregions: nasal-superior (NS), nasal (N), nasal-inferior (NI), temporal-inferior (TI), temporal (T), and temporal-superior (TS) (7–12) were measured by the SD-OCT in a circle with a distance of 3.5 mm to the optic nerve. Finally, both the BMO area (13) and HRT optic disc size (14) were exported. The main predictors regarding the diagnosis were, in descending order of importance, RNFL in the TI quadrant, vertical CDR, global BMO-MRW, and disc area. Due to the difference in age structure between eyes with a POD and GOD, age was excluded as a datapoint.

### 2.3. Machine Learning Workflow

**Figure 1:**
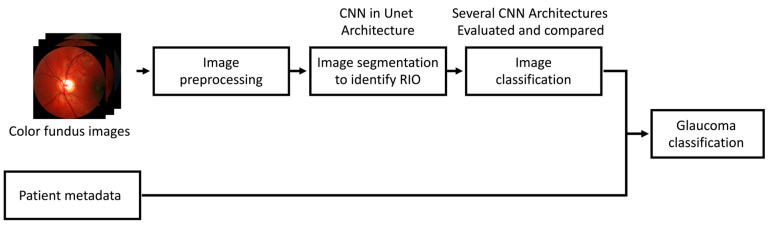
Machine learning (ML) workflow. Figure 1 shows a high-level overview of the entire ML process we used to classify patients. Several different algorithms for both image classification and glaucoma diagnoses were used. Legend: ROI = region of interest, CNN = convolutional neural network, U-Net = convolutional networks for biomedical image segmentation.

A two-step approach using fundus images only and a hybrid approach of fundus images combined with the metadata were used, as seen in Figure 1.

**Figure 2:**
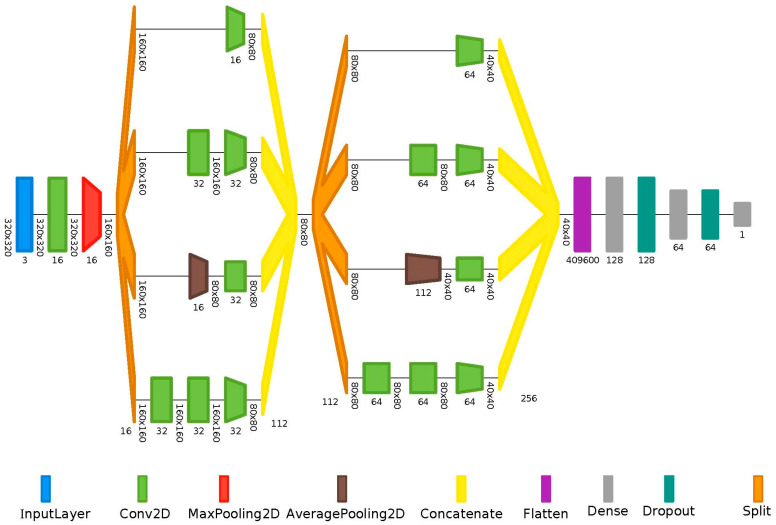
Inception convolutional neural network (CNN) architecture.

After image pre-processing to make the images uniform in size, we applied automated image segmentation to identify the region of interest (ROI, i.e., the area around the optic disc). For this purpose, we employed a convolutional neural network (CNN) with convolutional networks for biomedical image segmentation (U-Net) architecture, as proposed by Ronneberger et al. [27]. The extracted image segments had a size of 320 × 320 pixels. To increase the number of training samples, we used data augmentation on the fundus images. Data augmentation involves applying various transformations to the existing data to create additional training examples, which helps to improve a model’s generalization performance. We use 90° rotations, shifts, and reflections as the data augmentation procedures. For the actual classification of the images, we evaluated different CNN architectures and finally chose an inception architecture, as illustrated in Figure 2.

Dropout is a regularization technique in DL in which random neurons are temporarily turned off during training to prevent overfitting. We selected and optimized the hyperparameters of the CNN automatically with the help of Keras Tuner.

Due to the relatively small number of images, we did not achieve excellent accuracy in the image classification, especially for the comparison of patients with primary open-angle glaucoma with glaucoma suspects. To increase accuracy, we extended the classification algorithm to include patient metadata, as shown in Figure 1. The scores from the image classification contributed to this classification as one additional data field.

### 2.4. Statistics

Statistical analyses were performed using SPSS (IBM SPSS Statistics 23.0; IBM, Armonk, NY, USA). Prism was used for descriptive statistics (Prism 7, GraphPad Software, Inc., San Diego, CA, USA). Microsoft Excel (Microsoft^®^ Excel^®^ for Mac 2011, 14.6.2; Microsoft^®^, Redmond, DC, USA) was used for data management. The sensitivity, specificity, precision, and accuracy of the MLA were calculated. Furthermore, the area under the curve (AUC) was calculated.

The metadata were analyzed using one-way ordinary ANOVA. The level of significance was *p* < 0.05.

## 3. Results

### 3.1. Demographic Data

We included 1168 eyes from 584 patients. There were no significant differences between the groups regarding visual acuity and spherical equivalent. The ages of the GSOD (35 ± 20 years) and POD (48 ± 19 years) groups were significantly different from the GOD group (67 ± 13 years) (*p* < 0.001 for both). Therefore, age as an additional metadata point was excluded from the algorithm.

As expected, the MD of the SAP for both the GSOD and POD groups without VF defects differed significantly (*p* < 0.001) compared to the eyes with GOD and VF defects. Eyes with a GSOD excavation had a significantly larger Bruch’s membrane opening area (BMO-A) (*p* < 0.001) compared to the POD and GOD eyes. A larger BMO-A also explains a lower but not pathologically reduced BMO-MRW, as it is distributed over a larger area. However, the global RNFL, as well as its individual subsectors, did not differ between healthy and GSOD eyes and was only significantly different (*p* < 0.001) for the GOD eyes. The metadata of the population are summarized in Table 2.

**Figure 3:**
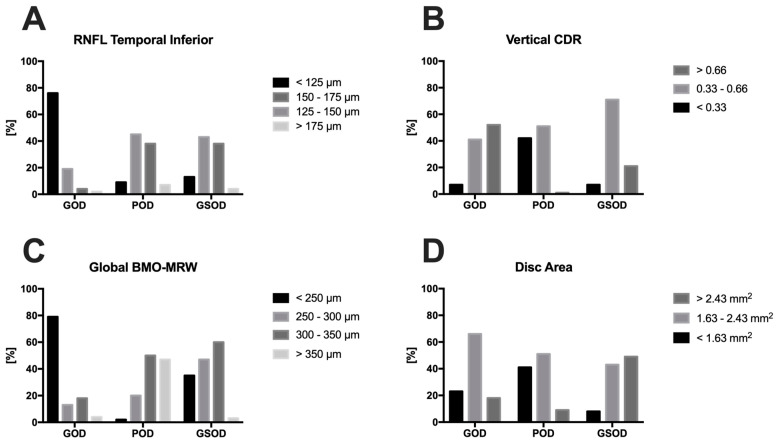
Distribution of the main predictors RNFL at the temporal inferior margin (**A**), the vertical Cup–Disc ratio (**B**), the global BMO-MRW (**C**), and the disc area (**D**) of the three groups analyzed. Legend: GOD = glaucomatous optic disc, POD = physiologic optic disc, GSOD = glaucomatous suspected optic disc, BMO-MRW = Bruch′s membrane opening minimal rim width, RNFL = retinal nerve fiber layer, CDR = cup–disc ratio.

The distribution of the RNFL at the TI margin of the optic disc, the vertical CDR, the disc area, and global MRW are seen in Figure 3 and represent a real-world scenario. The thickness of both the RNFL at the TI margin and the BMO-MRW were evenly distributed in patients with physiological and glaucoma-suspicious ONHs. The GSODs showed a similar distribution of CDR compared to the GODs. This was usually due to the fact that these are macrodiscs, which is why the optic disc area was enlarged compared to the GODs and healthy optic discs.

### 3.2. Performance of the Algorithm

The classification of glaucomatous and glaucoma-suspected ONHs (GOD vs. GSOD) and glaucomatous and physiological ONHs (GOD vs. POD) was performed in the first step (based on the color fundus photographs only) with a sensitivity, specificity, and accuracy of more than 75% each, as seen in Table 3. The best results, which are presented here, were achieved using an inception architecture.

By combining the images and metadata, the above parameters were increased to over 82% each. The best performance was achieved using a gradient boosting decision tree, as seen in Table 4.

## 4. Discussion

In this study, an inception CNN approach was able to automatically and reliably discriminate between eyes with GOD and GSOD and between GOD and POD using color fundus photographs. It was especially remarkable that the algorithm was able to differentiate between suspected and confirmed glaucoma on fundus photographs alone with good performance, which is a major challenge in clinical practice. Furthermore, CNN was able to improve this performance and achieve excellent results using a hybrid ML approach combining the images with metadata.

The strengths of the present study are as follows. First, the annotation of the data was based on the decision performed in clinical practice by glaucoma experts in a tertiary ophthalmic center, which leads to a high ground truth for the dataset used. The higher the ground truth, the better an algorithm can support ophthalmologists in clinical practice.

Second, the dataset included not only images and metadata from healthy or glaucoma patients but also from patients with suspected glaucoma (discs with cupping due to optic disc anomalies or macrodiscs), which corresponds to the daily clinical routine.

Third, by using a hybrid approach, data augmentation, and dropout methods, problems associated with a relatively small dataset were successfully avoided. Due to the methodology used in the present work, we believe that the generated algorithm could serve as a safety net mechanism to minimize the risk of misclassification and incorrect diagnosis and could, therefore, assist ophthalmologists in everyday clinical practice.

Using subjective grading of fundus images only for data annotation—in contrast to a full glaucoma workup—is a time-saving, low-cost option that allows huge amounts of data to be processed [17,28,29]. Li et al. [28] labeled 48,116 color fundus images as “referable” (yes vs. no) for glaucoma based on human graders, achieving an AUC, sensitivity, and specificity of 0.986, 95.6%, and 92%, respectively.

However, the approach of training DL models to replicate human grading of fundus photographs for glaucoma raises numerous potential problems. The agreement, even among experts, on the detection of ONH damage from fundus photographs is only moderate [30,31,32] and is known to have relatively poor reliability [33]. Furthermore, the assessment of the excavation to determine whether glaucoma exists based solely on fundus photographs is inaccurate [34,35]. Ophthalmologists tend to undercall glaucoma in small optic discs but overcall it in physiologically enlarged cups [28]. Thus, if human graders are used as the reference standard, the algorithms can only perform as well as the human graders and will essentially learn to replicate these common mistakes. Therefore, when the annotation of the data is based on abnormalities of the ONH on fundus images solely, such as a large excavation or large CDR [17,28,29], then no conclusions can be drawn regarding a secure glaucoma diagnosis [34,35]. Consequently, these algorithms can only distinguish between likely glaucoma and not glaucoma. Accordingly, algorithms that are based on subjective grading of the data on fundus images solely are suitable for glaucoma screening purposes only [36]. Nevertheless, these algorithms can be used to screen large patient groups for retinal diseases. A commercially available AI software (EyeArt, v2.2.0, Eyenuk, Inc., Los Angeles, CA, USA) recently received Conformité Européenne (CE) approval and can be used for the detection of diabetic retinopathy, age-related macular degeneration, and glaucomatous optic nerve damage in clinical practice [37].

In contrast, the methodology used in this study makes the proposed algorithm applicable beyond glaucoma screening in clinical practice. The labeling of the dataset was based on the diagnosis made by a glaucoma specialist in a tertiary eye care referral center in Germany after performing a clinical assessment on the patient and having access to a full glaucoma workup. Thus, a high ground truth could be achieved, which is one strength of the current study.

Here, the amount of data used in this study is comparable to other studies with similar methodologies. Noury et al. [38] included 291 normal eyes and 363 glaucomatous eyes with glaucomatous disc changes on fundus examination, with localized defects on OCT deviation or sector maps that correlated with VD defects that fulfilled the minimum definition of a Hodapp–Anderson–Parrish glaucomatous VF defect and had IOP-lowering treatment as per the chart review. The study by Medeiros et al. [39] stands out because they used a large amount of data with a high ground truth. The authors created a dataset consisting of 8831 eyes of 5529 glaucoma patients or glaucoma suspects that had a comprehensive clinical examination, including gonioscopy, funduscopy, OCT, and VF testing.

Another problem that arises from the subjective grading of images is the assessment of patients with suspected glaucoma. During a glaucoma consultation, it is particularly difficult even for an experienced ophthalmologist to distinguish between healthy and diseased patients, especially in the early stages of the disease, when glaucoma is first suspected. Therefore, the algorithm must ideally be trained with data from the mentioned patient group. Generating such a dataset is particularly challenging, and different approaches to generate datasets of glaucoma suspects to use for training a DL or ML algorithm have been described. Li et al. [40] used manual segmentation of fundus photographs and classified them as glaucoma suspect if any of the following criteria were present: a CDR over 0.7 and under 0.9, a rim width between 0.1 and 0.05, an RNFL defect, or disc hemorrhage. The methodologies used by Bhuiyan et al. [41,42,43], Atalay et al. [41,42,43], and Li et al. [41,42,43] similarly used the CDR as a reference for glaucoma suspects. Seo and Cho [25] used a DL classification of early normal-tension glaucoma and glaucoma suspects using BMO-MRW and RNFL. Glaucoma suspects were patients with suspicious clinical features who were not conclusive for glaucoma, including suspicious optic disc or RNFL changes; significant systemic, ocular, or family risk factors for glaucoma; or suspicious VF results but IOP within normal limits. Grewal et al. [44] classified 100 eyes based on optic disc examinations and divided them into normal (*n* = 35), glaucoma suspects (*n* = 30), and glaucoma (*n* = 35) eyes. Based on a study of 679 eyes, Yu et al. [45] defined glaucoma-suspected eyes as eyes with normal VF test results with any of the following criteria: IOP of 22 to 30 mmHg, asymmetric ONH cupping, or abnormal ONH appearance, or an eye that was the contralateral eye of unilateral glaucoma.

In our study, patients who were referred to our institute by general ophthalmologists as glaucoma suspects based on the optic disc appearance and were later judged as healthy were classified as GSOD. None of the glaucoma suspects received anti-glaucomatous medication or had a history of an IOP > 21 mmHg, and no VF defects were present. We believe that our methodology is very objective, and as the dataset consists of healthy patients and patients with mild, moderate, and severe glaucoma (see Figure 3) with a wide range of CDRs and RNFL defects, it corresponds to the clinical reality in terms of glaucoma.

However, our time-consuming methodology comes at the expense of the amount of data. One risk which is associated with the use of a small dataset is overfitting. This can occur if the model is trained with only a few images or too many training steps. In other words, the model learns patterns specific to the training data, which are irrelevant to other data. Thus, the performance of the algorithm when applied to new unknown data (e.g., in a clinical setting) is worse.

To increase the generalizability of the trained algorithm and avoid overfitting, two strategies were applied. First, we applied data augmentation to the fundus images, such as horizontal flipping and random cropping surrounding the ONH center. These data augmentation techniques increased the amount and diversity of fundus images within the training data, significantly improving our model’s performance and generalizability. Second, early stopping was applied. The selection of the number of steps to complete the training process was stopped when the absence of further improvement in the performance of the validation dataset occurred.

Nevertheless, the results and amount of data used in this study are comparable to those in the literature on glaucoma suspects. Seo and Cho [25] were able to discriminate between 229 eyes from glaucoma suspects and 168 from patients with normal tension glaucoma (NTG) with a CNN model using BMO-MRW and RNFL, achieving an AUC of 0.96. In a more recent work by the same research group, they achieved an AUC of 0.94, discriminating between 255 glaucoma suspects and 245 patients with NTG with a CNN model using BMO-based optic disc photography [46].

Similarly, in our study, for discriminating GOD from POD and GOD from GSOD on fundus images solely, robust AUCs of 0.88 and 0.84, respectively, were achieved. Due to the relatively small number of images, the accuracy we achieved with the image classification was not excellent, especially for the comparison of POAG patients with glaucoma suspects. To increase accuracy, we extended the classification algorithm to include patient metadata. For an algorithm that is intended for screening large numbers of patients, the use of more than one data source (e.g., fundus images) is not expedient. However, since the proposed algorithm is designed to support ophthalmologists in everyday clinical practice, it can be assumed that various metadata are collected and used in the decision-making process, as this leads to better-informed decision making and improved patient outcomes. Similarly, we achieved an excellent AUC of 0.99 for discriminating GOD from POD and 0.92 for discriminating GOD from GSOD using both fundus images and metadata.

### Limitations

There are several limitations to this work. First, we used a relatively small dataset. However, this could not be avoided due to the chosen methodology and the associated time-consuming data annotation. Nevertheless, problems such as overfitting were circumvented. Second, other optic nerve diseases besides glaucoma were not included. Therefore, the algorithm can only be used for glaucoma diagnostics.

Third, despite the expected very high ground truth in the dataset used, it could be further optimized. Thus, a dataset that is based only on data from patients who have been monitored over a period of, for example, five years would make the diagnosis even more reliable.

Fourth, a detailed, time-consuming examination of the patient using different imaging modalities and automated data export to make the data accessible to the algorithm is necessary. This means that the algorithm will only be able to be implemented by a very small number of users.

Since a misdiagnosis by the algorithm could lead to blindness in the patient, it is necessary to demonstrate the non-inferiority of the algorithm in comparison to a glaucoma expert before approval in clinical practice. Ultimately, the algorithm is designed to support ophthalmologists in everyday practice and not to replace them.

## 5. Conclusions

In this study, we used a carefully selected, large European dataset based on the diagnosis made by a glaucoma expert at a tertiary care clinic and achieved a high ground truth. The dataset consisted of patients with POD, GOD, and especially GSOD, reflecting a real-world scenario in terms of glaucoma. Despite the relatively small dataset in the context of ML and AI, the algorithm generated in this work was able to robustly distinguish between patients with GOD, GSOD, and POD on fundus images solely and reached excellent performance using additional metadata. With the help of the algorithm, ophthalmologists could be supported in their everyday practice, and the number of co-assessments at a tertiary center could be reduced.

## Figures and Tables

**Table 1:** Distribution of the annotated data population.

Group	1 = GOD	2 = GSOD	3 = POD
*n*	336	310	321

*n* = number, GOD = glaucomatous optic disc, GSOD: glaucoma-suspected optic disc, POD: physiologic optic disc.

**Table 2:** Metadata of the study population used.

Group	GOD (1)	GSOD (2)	POD (3)	1 vs. 2 *p*	1 vs. 3 *p*	2 vs. 3 *p*
Value	M ± SD	M ± SD	M ± SD			
*n*	336	310	321			
Age (years) *	67 ± 13 69 (62; 78)	35 ± 20 28 (18; 53)	48 ± 19 48 (29; 59)	<0.001	<0.001	>0.05
Visual acuity (decimal)	0.68 ± 0.30 0.70 (0.50; 0.80)	0.90 ± 0.20 1.00 (0.80; 1.00)	0.81 ± 0.27 0.80 (0.63; 1.00)	>0.05	>0.05	>0.05
Spherical equivalent (dpt.)	−0.81 ± 2.00 −0.38 (−2.00; 0.50)	−0.61 ± 2.48 0.00 (−1.60; 0.60)	−0.44 ± 2.38 −0.13 (−1.00; 0.75)	>0.05	>0.05	>0.05
MD (dB)	−7.96 ± 9.82 −4.62 (−12.52; −1.28)	−1.01 ± 2.15 −0.77 (−1.99; 0.39)	−3.34 ± 6.41 −1.41 (−3.52; 0.24)	<0.001	<0.001	>0.05
CDR horizontal	0.66 ± 0.20 0.67 (0.55; 0.80)	0.56 ± 0.16 0.59 (0.50; 0.66)	0.33 ± 0.20 0.36 (0.20; 0.45)	>0.05	0.01	>0.05
CDR vertical	0.68 ± 0.20 0.69 (0.55; 0.85)	0.53 ± 0.24 0.55 (0.50; 0.61)	0.32 ± 0.20 0.33 (0.20; 0.43)	0.03	0.002	>0.05
BMO area (mm^2^)	1.97 ± 0.43 1.94 (1.68; 2.27)	2.57 ± 0.57 2.52 (2.21; 2.90)	1.93 ± 0.43 1.88 (1.68; 2.16)	<0.001	>0.05	<0.001
Global BMO-MRW (µm)	197 ± 77 192 (141; 239)	264 ± 40 261 (239; 288)	352 ± 68 347 (305; 389)	<0.001	<0.001	<0.001
Global RNFL (µm)	69 ± 19 69 (54; 82)	96 ± 11 98 (90; 104)	99 ± 10 100 (92; 105)	<0.001	<0.001	>0.05
NS (µm)	78 ± 28 77 (57; 98)	108 ± 23 109 (93; 125)	113 ± 22 113 (98; 127)	<0.001	<0.001	>0.05
N (µm)	59 ± 19 60 (47; 72)	80 ± 13 80 (72; 89)	81 ± 14 81 (71; 91)	<0.001	<0.001	>0.05
NI (µm)	78 ± 27 76 (57; 96)	110 ± 26 112 (94; 127)	110 ± 24 109 (94; 126)	<0.001	<0.001	>0.05
TI (µm)	91 ± 40 87 (58; 123)	143 ± 23 146 (133; 158)	151 ± 56 147 (137; 160)	<0.001	<0.001	>0.05
T (µm)	54 ± 17 53 (42; 66)	68 ± 10 69 (61; 75)	70 ± 12 70 (62; 76)	<0.001	<0.001	>0.05
TS (µm)	88 ± 34 87 (58; 113)	127 ± 23 129 (114; 144)	134 ± 20 135 (122; 147)	<0.001	<0.001	>0.05
Disc area (mm^2^)	1.89 ± 0.42 1.83 (1.63; 2.14)	2.37 ± 0.80 2.43 (2.07; 2.73)	1.80 ± 0.49 1.79 (1.48; 2.13)	<0.001	<0.05	<0.001

Legend: *n* = number included; CDR = cup–disc ratio; BMO-MRW = Bruch’s membrane opening minimum rim width; RNFL = retinal nerve fiber layer; M = mean; NS = nasal-superior; N = nasal; NI = nasal-inferior; TI = temporal-inferior; T = temporal; TS = temporal-superior; * excluded from metadata.

**Table 3:** Results of image classification.

ML Performance	TP	TN	FP	FN	Sn	Sp	Acc.	Pr.	F1	AUC
GOD vs. POD	59	47	17	5	92%	73%	83%	78%	84%	0.88
GOD vs. GSOD	48	47	15	14	77%	76%	77%	76%	77%	0.84

Legend: TP = true positive, TN = true negative; FP = false positive; FN = false negative; Sn = sensitivity; Sp = specificity; Acc = accuracy; Pr = precision; F1 = F1 score, GOD = glaucomatous optic disc; POD = physiologic optic disc; GSOD = glaucomatous suspected optic disc; AUC = area under the curve.

**Table 4:** Results of the classification based on metadata and image scores using an inception convolutional neural network (CNN) architecture.

ML Performance	TP	TN	FP	FN	Sn	Sp	Acc.	Pr.	F1	AUC
GOD vs. POD	60	60	4	4	94%	93%	94%	94%	94%	0.99
GOD vs. GSOD	52	56	6	10	84%	90%	87%	89%	86%	0.92

Legend: TP = true positive, TN = true negative; FP = false positive; FN = false negative; Sn = sensitivity; Sp = specificity; Acc = accuracy; Pr = precision; F1 = F1 score; GOD = glaucomatous optic disc; POD = physiologic optic disc; GSOD = glaucomatous suspected optic disc; AUC = area under the curve.

## Data Availability

The raw data supporting the conclusions of this article will be made available by the authors on request.
